# Investigation of Enterogermina’s Protective and Restorative Mechanisms on the Gut Microbiota with PPI, Using SHIME Technology

**DOI:** 10.3390/nu15030653

**Published:** 2023-01-27

**Authors:** Cindy Duysburgh, Lynn Verstrepen, Mattia Van den Broeck, Zefferino Righetto, Marcos Perez

**Affiliations:** 1 ProDigest BV, 9052 Zwijnaarde, Belgium; 2 Sanofi, Industriepark Hoechst, 65929 Frankfurt, Germany

**Keywords:** *Bacillus clausii*, dysbiosis, probiotic, Enterogermina, gut microbiome, metagenomics, microbiota, omeprazole, proton pump inhibitor, SHIME^®^

## Abstract

Proton pump inhibitors (PPIs) are commonly prescribed medications associated with changes in the gut microbiome and dysbiosis when used long-term. Probiotics, such as Enterogermina^®^ (containing four strains of *Bacillus clausii*) reduce side effects from triple therapy with PPI+antibiotics. We aim to assess the ability of this probiotic in preventing and/or treating the dysbiosis induced by PPI use. Faecal samples from six healthy donors were used to colonise a Triple-Mucosal-Simulator of the Human Intestinal Microbial Ecosystem^®^ model with added ileal compartment. Changes in the microbial community composition and metabolite production were measured for PPI alone (control), PPI+Enterogermina (preventative), and Enterogermina treatment after PPI (curative). Differences were assessed by one-way ANOVA with Tukey’s multiple comparisons test. The model was shown to replicate some of the effects of long-term PPI use. There were significant changes in microbial diversity and an increase in butyrate levels in the preventative and curative arms, indicative of a beneficial effect to gut health. Probiotic use countered some of the effects of PPI use: *Streptococcus bovis* levels increased in the control arm but reduced following probiotic treatment. These results show that probiotic treatment with *B. clausii* may have beneficial effects on the gut microbiota following PPI treatment.

## 1. Introduction

Proton pump inhibitors (PPIs) are commonly prescribed medications [1]. They reduce stomach acid production by irreversibly inhibiting the H^+^/K^+^ ATPase proton pumps [2]. Although proven to be well-tolerated, long-term PPI use can cause vitamin B12 deficiency, hypomagnesemia, and *Clostridium difficile* infections, among other side effects [3]. Recent studies have investigated the association between long-term PPI use and small intestinal bacterial overgrowth (SIBO), with some studies indicating a negative influence on health due to PPI-induced dysbiosis [4,5,6]. Studies have also suggested that short-term (~2 weeks) PPI use does not extend a preventative effect to the small intestine in the case of non-steroidal anti-inflammatory drug (NSAID)-associated small intestine injury; >50% of subjects exhibited such damage following short-course NSAID+PPI use [7]. Use of PPIs short-term may exacerbate NSAID-induced intestinal damage in part by inducing significant shifts in enteric microbial populations [8]. Furthermore, PPI intake has been associated with an increase in the levels of potentially pathogenic bacteria such as *Enterobacteriaceae*, *Enterococcaceae,* and *Staphylococcaceae* in the small intestine and colon [9] and an increased risk of community-acquired enteric infections, especially those caused by *Salmonella* and *Campylobacter* spp [10].

Enterogermina^®^ (Sanofi) is a probiotic comprising four strains of *Bacillus clausii* (O/C, N/R, SIN, and T) that has been used effectively and safely for several decades [11]. Enterogermina was used as an adjunct to triple therapy (i.e., alongside two antibiotics and a PPI) in the treatment of *Helicobacter pylori* infections [11] and has been shown to reduce the adverse effects, such as diarrhea, nausea, and pain, which were largely attributed to the antibiotic component of the therapy [12]. Even though *B. clausii* has been in use for decades, several of its mechanisms of action have only been described in recent years, in the context of rotaviral infections and as a probiotic with antimicrobial and immunomodulatory activity [13,14,15].

We theorised that the beneficial effects seen with *B. clausii* use as an adjunct to triple therapy may also be more broadly applicable in PPI use. In this study, we aimed to assess the potential ability of this probiotic in preventing and/or treating the dysbiosis induced by chronic treatment with PPIs. For this purpose, the in vitro Triple-Mucosal-Simulator of the Human Intestinal Microbial Ecosystem^®^ (Triple-M-SHIME) model, which simulates the different regions of the gastrointestinal tract (including a newly developed ileal compartment containing a standardized ileal microbial consortium) was used. Using this model, we investigated community-level shifts in the microbial ecosystem using quantitative 16S rRNA sequencing and changes in metabolic activity. Based on this exploratory study, we present key findings related to PPI-induced dysbiosis and present a post hoc hypotheses regarding the mechanism of action of *B. clausii* in this context.

## 2. Materials and Methods

### 2.1. Donor Pre-Screening

Faecal samples were collected from six healthy donors with no known history of gut diseases and no antibiotic use in the three months prior to sampling. The faecal samples were collected in a sampling box together with an Anaerogen bag to remove all oxygen from the environment. Anaerobic phosphate buffer (containing 8.8 g/L K_2_HPO_4_, 6.8 g/L KH_2_PO_4_, 0.1 g/L sodium thioglycolate and 0.015 g/L sodium dithionite) was used to prepare a faecal slurry, which was then centrifuged for two minutes at 500× *g* to remove large particles. The resulting supernatant was mixed with an equal volume of cryoprotectant solution, prepared as described in Hoefman S, et al. [16]. This mixture was homogenized, snap-frozen in liquid nitrogen, and stored at −80 °C. To select a donor whose faecal sample could be used to inoculate the colonic environments used in this study, quantitative polymerase chain reaction (qPCR) analyses were conducted to determine levels of *Enterobacteriaceae* according to methods published previously [17]; higher levels of these bacteria would be representative of PPI-induced dysbiosis.

### 2.2. SHIME Reactor Setup

**Figure 1:**
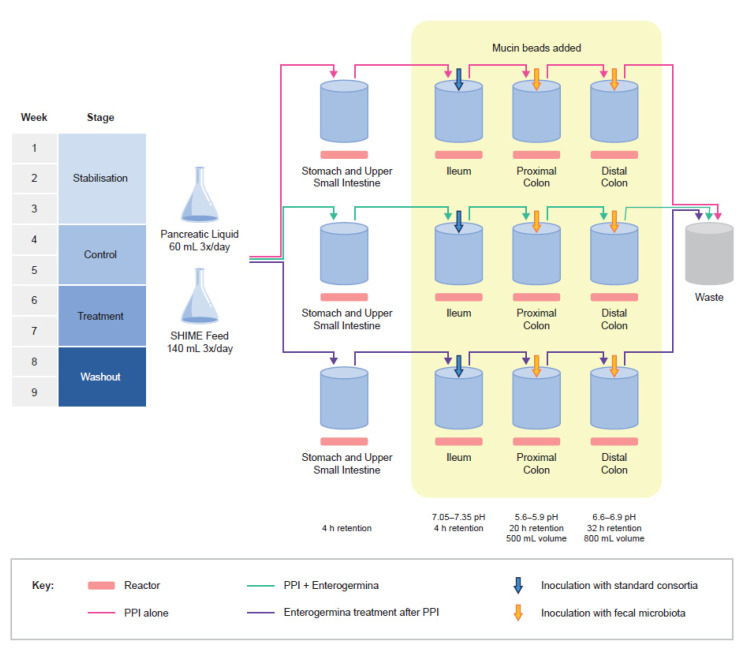
The adapted design of the Triple-M-SHIME^®^ used in this study. This adapted design comprised a combined stomach + small intestine reactor with retention times and pH ranges optimised to represent a full GI tract simulation, an ileal region inoculated with the ileal consortium, two colon regions (proximal and distal) rather than three, as a compromise for the additional test conditions, inoculated with the donor-derived faecal consortium, and mucin beads added to the ileal and colonic environments, allowing the sampling of mucosa- and lumen-associated microbiota. Each reactor was subjected to a stabilisation, control, treatment, and washout period over nine weeks of the study duration. Mucin beads were added to the ileal and colonic reactors to replicate the mucosal environment of the intestine. Samples from the luminal and mucosal environment were collected during each stage from each reactor.

The typical setup of the SHIME reactor has been described previously [18]. Briefly, a five-stage reactor simulates the human gastrointestinal tract: the first two reactors represent the stomach and small intestine, and the last three reactors represent the ascending, transverse, and descending colons. This setup only represents the microbial community present in the lumen and excludes the mucosal ecosystem. Therefore, the Triple-M-SHIME reactor setup was used during this specific study (Figure 1) to allow the culture of both luminal and mucosa-associated microbes over several weeks [19], whereby the mucosal environment is simulated using mucin-covered beads. In addition, the ileal SHIME was developed using a proprietary, highly specific composition representative of the ileal microbial community.

### 2.3. Adapted SHIME Setup in this Study

The Triple-M-SHIME investigation consisted of four stages (Figure 1). During the stabilisation period, the ileal reactors were inoculated with the ileal consortium, and the colon reactors were inoculated with the cryopreserved faecal sample from the selected donor. This was followed by a three-week period during which a basic nutritional matrix was provided to allow the microbial communities to become established in the local environment of the reactors. Components of the basic nutritional matrix were supplied by Sigma-Aldrich, unless otherwise stated. The ileal nutritional medium was composed of 1.9 g/L bile salts (BD Bioscience, CA, USA), 0.25 g/L special peptone (Oxoid, Basingstoke, UK), 0.74 g/L yeast extract (Oxoid, Basingstoke, UK), 0.49 g/L mucin (Carl Roth, Karlsruhe, Germany), 0.12 g/L L-cysteine-HCl, and 1.1 mL/L Tween 80. To this solution, a filter-sterilized stock solution (5% *v*/*v*) was added containing 50 g/L NaHCO_3_ (Chem-Lab, Zedelgem, Belgium), 10 g/L NaH_2_PO_4_ (VWR, PA, USA), 10 g/L K_2_HPO_4_ (Chem-Lab, Zedelgem, Belgium), 0.9 g/L MgSO_4_∙7H_2_O (Chem-Lab), 0.5 g/L MnCl_2_∙4H_2_O (VWR), 0.9 g/L CaCl_2_∙2H_2_O (VWR), 0.05 g/L FeSO_4_∙7H_2_O, 0.05 g/L ZnSO_4_∙7H_2_O, 0.05 g/L hemin, 4 g/L glucose, 4 g/L fructose, 4 g/L sucrose, 4 g/L maltose (Carl Roth, Karlsruhe, Germany), 4 g/L lactose (Oxoid, Basingstoke, UK), 2.5 g/L mannose (Carl Roth, Karlsruhe, Germany), and 2.5 g/L galactose. The colonic reactors received an additional influx of fibre solution containing 3.4 g/L arabinogalactan (Keyser & Mackay, OH, USA), 5.6 g/L pectin (Keyser & Mackay, OH, USA), 1.4 g/L xylan (Carl Roth, Karlsruhe, Germany), 11.2 g/L starch (Carl Roth, Karlsruhe, Germany), 1.12 g/L glucose, 2.1 g/L special peptone (Oxoid, Basingstoke, UK), 6.3 g/L yeast extract (Oxoid, Basingstoke, UK), 4.2 g/L mucin (Carl Roth, Karlsruhe, Germany), and 1.0 g/L L-cysteine-HCl.

During the two-week control period (C), basic nutritional matrix supplementation was continued. Samples collected in this period were used to determine the baseline microbial community composition and activity as a reference for the treatment arms. Sampling frequency is described separately for each experiment in the sections below. During the two-week treatment period (TR), the reactors were supplemented with the PPI omeprazole (14 mg/day added to the ileum to closely mimic repeated intake of PPIs) under the following conditions: (1) PPI alone (PPI control); (2) simultaneous PPI + Enterogermina (preventive); and (3) PPI for 2 weeks followed by Enterogermina (curative). Enterogermina was administered at a daily dose of 6 × 10^9^ colony-forming units in the preventive and curative arms. During the two-week washout period (WO), PPI treatment was withdrawn from all arms, and Enterogermina supplementation was withdrawn from arm 2 and initiated in arm 3 at the same dose as was used in the preventive arm.

Because PPI treatment shifts the gastric environment to a pH that is optimal for α-amylase activity (from pH 2.0 to > pH 6.0) [20], PPI use is expected to increase the levels of disaccharides and trisaccharides in the small intestine. To replicate this effect in the Triple-M-SHIME setup, additional sugars and proteins were administered from the treatment period onwards: the additional sugars added to the stock solution included glucose, fructose, sucrose, maltose, and lactose, at a final colon concentration of 0.15 g/L each, and mannose and galactose at a final colon concentration of 0.09 g/L during the control period, all of which were doubled during PPI administration; the additional proteins added to the ileal nutritional medium included casein, albumin, and meat peptone at a final colon concentration of 1 g/L each.

Variance in the levels of short-chain fatty acid (SCFA) levels were used as a marker of the stability of the microbial community in the Triple-M-SHIME reactor, as previously reported [21].

### 2.4. Microbial Community Composition

#### 2.4.1. qPCR Analysis of Enterogermina Engraftment

Samples were collected from the luminal and mucosal environments three times each during the final week of the C, TR and WO periods. A qPCR protocol was used to determine the successful establishment of the *B. clausii* strains in the probiotic in these environments. The hexadecyltrimethylammonium bromide (CTAB)-phenol-chloroform method was used to isolate DNA from the samples. The *erm34* gene was amplified using species-specific primers (Table 1) designed by ProDigest based on previous literature [22]. The cycling conditions were as follows: initial denaturation at 95 °C for 10 s; 40 cycles of denaturation at 95 °C for 15 s, annealing at 60 °C for 30 s, extension at 72 °C for 30 s; and a final melt curve of 95 °C for 15 s, 60 °C for 1 m, and 95 °C for 15 s.

#### 2.4.2. Quantitative 16S rRNA Sequencing

The luminal and mucosal environments were sampled three times in the final week of the C, TR, and WO periods. To identify and classify the bacterial species in each treatment arm, 16S rRNA sequencing was conducted. The proportional abundance of different taxa at different phylogenetic levels (phylum-, family-, and operational taxonomic unit [OTU]- levels) were determined using primers that spanned two hypervariable regions (V3–V4) of the 16S gene (Table 1). These were adapted from previously reported primer sequences [23]. A paired sequencing approach was used; 2 × 250 bp results were sequenced in 424 bp amplicons. Flow cytometry was used to determine the numbers of total bacterial cells in the samples [24]. By combining the cell count data with the phylogenetic information from 16S rDNA sequencing as descried previously, accurate quantitative information of the different taxonomic entities was obtained [25].

#### 2.4.3. Shifts in Microbial Community Composition

Shifts in microbial community composition were investigated in the ileal and colonic environments in each arm at the phylum-, family-, and OTU-level. PPI treatment was considered to have induced dysbiosis if the levels of a phylum, family, or OTU in the TR stage of the PPI control arm were significantly different from those in the C stage of the PPI control arm. Probiotic treatment was deemed as having countered this dysbiosis if the levels of that phylum, family, or OTU in the TR stage of the preventive arm or WO stage of the curative arm were significantly different from the corresponding stages in the PPI control arm, but not significantly different from the C stage of the same arm. Cross-feeding effects due to probiotic treatment were considered if changes in OTU levels coincided with probiotic treatment, i.e., significantly different levels in the TR stage of the preventive arm or the WO stage of the curative arm when compared with the corresponding stages of the PPI control arm.

### 2.5. Microbial Community Activity and Fermentative Activity

SCFA and branched SCFA levels were measured as described previously [26]; butyrate, acetate, and propionate concentrations were measured to determine the differences in carbohydrate metabolism in the different treatment arms. Lactate levels were also measured using a commercially available enzymatic assay kit (R-Biopharm, Darmstadt, Germany), according to manufacturer’s instructions. Ammonium analysis was performed using a KjelMaster K-375 device (Büchi, Hendrik-Ido-Ambacht, The Netherlands). Branched SFCA and ammonium levels were taken as markers of proteolytic fermentation. Acid–base consumption was measured to assess the production of metabolites by the colonic microorganisms.

### 2.6. Statistics

All ileal and colonic samples were biological replicates collected on different days of the week. During the two-week C period, three samples were collected per week, resulting in six samples per reactor. In the TR and WO periods, three samples were collected only in the final week, resulting in three samples each for the TR and WO reactors in each arm.

For qPCR analyses, three technical replicates from each biological replicate were used. Biological replicates were independent sampling points that represented biologically meaningful variation, whereas technical replicates came from a single sample and represented the precision of experimental techniques.

For microbial community composition experiments, data were log-transformed to convert them into a normal distribution. Data points below the limit of quantification (LOQ) were replaced with the value of the LOQ to allow for statistical comparisons. The reciprocal Simpson diversity index was used to measure diversity in terms of species richness and evenness [27]. This index considers species richness and evenness. Higher diversity (higher number of species that are evenly distributed) is reflected by higher index numbers, i.e., a community with only one OTU would have a diversity index of 1.

Differences between the TR, WO, and C stages within a study arm were evaluated using a one-way ANOVA with Dunnett’s multiple comparisons test. Differences between the PPI control, preventive, and curative arms were evaluated using a one-way ANOVA with Tukey’s multiple comparisons test. Because we had no prior hypotheses concerning the magnitude or direction of changes due to the different treatments, the statistical comparisons were not powered to detect these changes; therefore, the significance conclusions are meant to be interpreted from an exploratory point of view. Exact *p*-values for all comparisons conducted are provided in the supplementary tables, and the most relevant comparisons are included in the body of this paper with the caveat that an alpha-significance level of *p* < 0.05 has been chosen without prior expectation.

## 3. Results

### 3.1. Donor Pre-Screening

**Figure 2:**
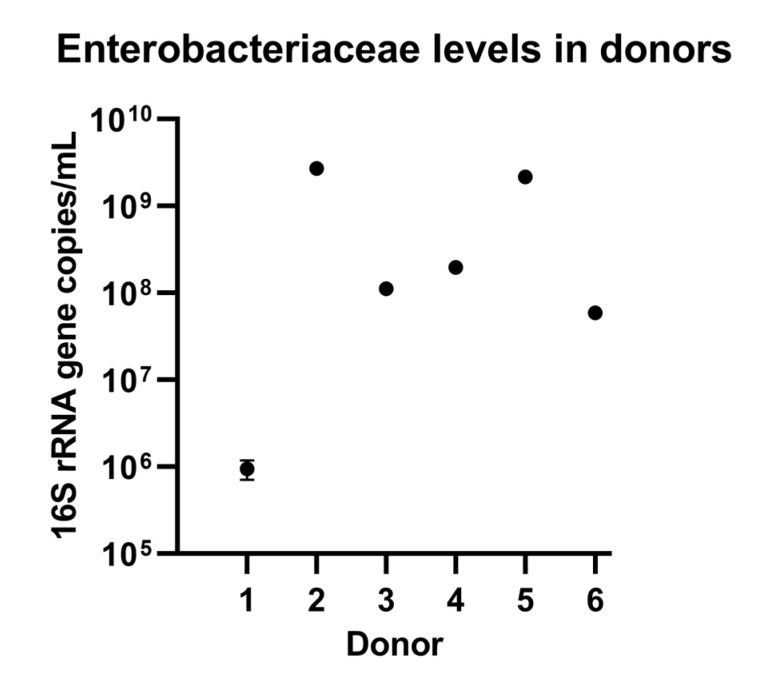
Enterobacteriaceae levels measured by 16S rRNA-specific qPCR revealed donors with high levels of this bacteria. Data from three technical replicates of the qPCR are presented as means±SD.

PPI intake correlates with high levels of *Enterobacteriaceae*; therefore, we pre-screened donors to select a faecal sample representative of PPI-induced dysbiosis for use as inoculum in the colonic environments. Two donors had high levels of these bacteria (Figure 2). From previous experiments using these samples, the faecal sample from Donor 5 was known to contain high levels of butyrate-producing species. Therefore, this faecal sample was used for inoculating the colonic environments in our Triple-M-SHIME setup.

### 3.2. Stability of the Adapted SHIME Setup

To determine the stability of the colonising microbial community, we monitored SCFA levels throughout the control period, which were found to be stable [21]. During the control period, an average variance of 5% was observed in SCFA levels within each reactor, and an average variance of 6.4% was observed between the control vessels of different treatment arms.

### 3.3. Microbial Community Composition

#### 3.3.1. *B. clausii* Was Successfully Established in the Three Regions of the SHIME Setup

**Figure 3:**
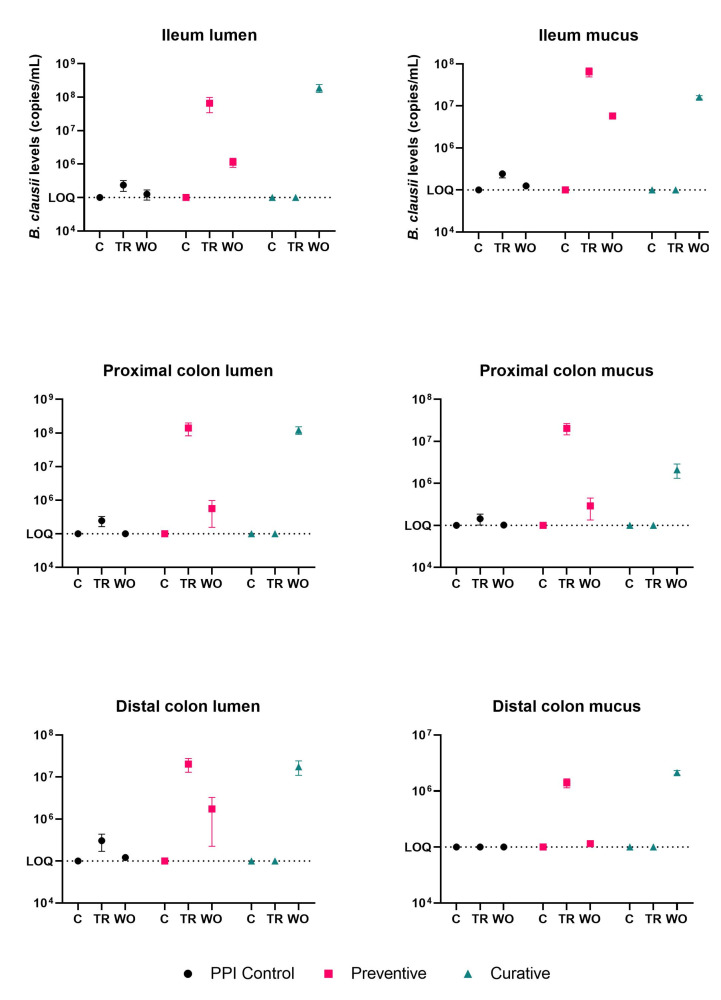
Average levels of *B. clausii* (copies/mL of the erm34 gene) in the different arms of the study. Means ± SDs are shown for three biological replicates.

We used species-specific qPCR to monitor levels of *B. clausii* aggregate through the different treatment stages in each arm. In the control (C) stage, *B. clausii* levels were below the limit of quantitation in all three arms (PPI control, preventive, and curative) in the ileum, proximal colon, and distal colon (Figure 3). In the treatment (TR) stage, *B. clausii* levels increased significantly (*p* < 0.001) in the lumen of the PPI control and preventive arms compared with the respective C stages, while remaining below detectable levels in the curative arm (Figure 3, Table S1). Similar trends were observed in the mucosal environment (Figure 3, Table S1). During the washout stage, *B. clausii* levels decreased significantly in the preventive arm when compared with the control and/or treatment stages (*p* < 0.001 in each case) and increased to significantly higher levels in the curative arm in the ileum, and the proximal and distal colon (*p* < 0.001 in each case; Figure 3). This indicates that the test strains survived and reproduced in the ileum and colon regions and were retained at levels higher than in the control stage in the luminal and mucosal environments.

#### 3.3.2. *B. clausii* Treatment Altered the Microbial Community Composition

##### Changes in the Ileal Community

In the ileal lumen in the PPI control arm, specific increases in levels of *Enterococcus faecalis* were observed: 8.46 log cells/mL in the TR stage (*p* = 0.021) and 8.55 log cells/mL in the WO stage (*p* = 0.015) compared with 7.28 log cells/mL in the C stage (Table S2). This points to the potential role of this species in the development of SIBO following PPI treatment (Table 2). However, probiotic treatment in the preventive and curative arms did not diminish levels of these species: 8.51 log cells/mL and 8.70 log cells/mL in the TR and WO stages, respectively, of the preventive arm; and 8.27 log cells/mL and 8.73 log cells/mL, respectively, in the TR and WO stages of the curative arm. These levels were not significantly different from the TR and WO stages of the PPI control. This indicates that *B. clausii* treatment at this dose would be insufficient in the control of SIBO if it were caused by this species (Table 2). These community shifts in the lumen were also replicated in the mucosal environment of the ileum (Table 3 and Table S2).

##### Changes in the Colonic Community: Alpha Diversity

Microbial diversity showed a downward trend in the lumen of the proximal and distal colon and the mucus of the proximal colon during the TR stage of the PPI control arm, even though these differences were not statistically significant when compared with the respective C stages of these arms. However, diversity increased significantly in the WO stage of the lumen of the proximal and distal colon and the mucus of the distal colon when compared with the C and TR stages, respectively (*p* < 0.05 in each case, exact *p*-values in Table S3 and Table 4).

Preventive treatment with the probiotic significantly increased diversity in the TR stage compared with the C stage of the same arm, both in the lumen (*p* = 0.037) and mucus (*p* = 0.013) of the distal colon, as well as when compared with the TR stage of the PPI control arm in the lumen and mucus of the distal colon (*p* < 0.001 in each case; Table 4 and Table S3) and in the lumen of the proximal colon (*p* = 0.004; Table 4 and Table S3).

Curative treatment significantly increased microbial diversity in the mucus of the distal colon during the WO stage when compared with both C and TR stages (*p* < 0.001 in each case; Table 4 and Table S3).

##### Changes in the Colonic Community: Phylum-Level Shifts

There were no statistically significant family-level changes in the lumen of the proximal colon after PPI treatment in the PPI control arm, with the exception of significantly increased levels of Synergistetes in the WO stage compared with the C and TR stages (*p* < 0.001 in each case; Table 5 and Table S4). A significant increase in Synergistetes levels was also seen in the WO stage of the preventive arm compared to the C and TR stages (*p* = 0.014 in each case; Table 5 and Table S4).

A significant increase in Synergistetes was also noted in the lumen of the distal colon in the TR stage compared with the C stage in the curative arm (*p* = 0.018), in the WO stage compared with the C stage in all three arms (*p* < 0.001 in PPI control, *p* = 0.036 in preventive and *p* = 0.004 in curative), and in the WO stage compared with the TR stage in the PPI control arm (*p* < 0.001; Table 6). In addition, the lumen of the distal colon had significantly higher levels of Desulfobacteria in the WO stage compared with the C stage of the curative arm, coinciding with probiotic treatment (*p* = 0.048; Table 6).

Levels of Verrucomicrobia significantly decreased in the TR stage compared with the C stage of the PPI control arm in the lumen of the distal colon (*p* = 0.03). However, levels of this phylum were largely unaffected by probiotic treatment, indicated by the levels of this phylum in the TR stage compared with the C stage of the preventive arm, and the significantly higher levels of this phylum in the WO stage compared with the TR stage (*p* = 0.02) of the curative arm (Table 6 and Table S4).

The mucosal environments of the proximal and distal colon were dominated by Firmicutes. Their levels were largely unaffected in the proximal colon, significantly decreased (*p* < 0.05) in the WO stage compared with the C stage in all arms, and significantly decreased in the WO stage compared with the TR stage in the PPI control (*p* = 0.007) and curative (*p* = 0.014) arms of the distal colon (Table 7, Table 8 and Table S4). This change in the mucus of the distal colon was accompanied by significant increases in levels of Actinobacteria in the WO stage of the PPI control (*p* = 0.033) and preventive arms (*p* = 0.002) compared with the respective C stages of these arms. The levels of Bacteroidetes also significantly increased in the WO stage of PPI control (*p* = 0.006 compared with C and *p* = 0.042 compared with TR) and curative arms (*p* = 0.014 compared with C and *p* = 0.026 compared with TR stage; Table 8 and Table S4).

##### Changes in the Colonic Community: Family-Level Shifts

In the lumen of the proximal colon, there were no family-level shifts that were significantly different between the TR and C stages of the PPI control (Table 9). Family level changes that co-occurred with probiotic treatment were seen in *Bacteroidaceae* and *Ruminococcaceae*, in addition to the expected change in *Bacillaceae*, of which *B. clausii* is a member (Table 9).

In the lumen of the distal colon, PPI treatment in the PPI control arm led to significant decreases in the levels of *Coriobacteriaceae* (*p* = 0.037) and *Selenomonadaceae* (*p* = 0.026) compared with the C stage of this arm (Table 9 and Table S5). These changes were rescued by probiotic treatment, as seen by the relatively similar levels of *Coriobacteriaceae* and *Selenomonadaceae* in the TR and C stages of the preventive and curative arms (Table 9 and Table S5).

Probiotic treatment coincided with a significant increase in the levels of *Enterococcaceae* in the preventive arm in the TR (*p* = 0.028) and WO stages (*p* < 0.001) compared with the C stage and in the WO stage compared with the TR stage (*p* = 0.002; Table 9 and Table S5) and in the curative arm in the WO stage compared with the C (*p* = 0.001) and TR stages (*p* = 0.015; Table 9 and Table S5).

Probiotic treatment also coincided with significantly increased levels of *Christensenellaceae* in the TR stage of the preventive arm compared with the C stage of the same arm (*p* = 0.043; Table 9 and Table S5) and in the TR (*p* = 0.017) and WO stages (*p* = 0.001) of the curative arm compared with the C stage of the same arm and in the WO stage compared with the TR stage of the curative arm (*p* = 0.025; Table 9 and Table S5).

In the mucus of the proximal colon, PPI treatment in the PPI control arm led to significant decreases in *Prevotellaceae* in the TR stage compared with the C stage (*p* = 0.001; Table 9 and Table S5); this was rescued by probiotic treatment as seen by the higher levels of this family in the TR stage of the preventive arm compared with the TR stage of the PPI control arm (*p* < 0.001; Table 9 and Table S5) and by the higher levels in the WO stage of the curative arm compared with the WO stage of the PPI control arm (*p* = 0.029; Table 9 and Table S5), though these increases in the TR stage of the preventive and WO stage of the curative arms were not significantly different from the respective C stages (Table 9 and Table S5).

PPI treatment also led to significant increases in *Streptococcaceae* levels in the TR stage of the PPI control compared with the C stage of this arm (*p* = 0.001; Table 9 and Table S5). This effect was rescued by probiotic treatment: the TR stage of the preventive arm was significantly different from the TR stage of the PPI control arm (*p* = 0.001), while not significantly different from the C stage of the preventive arm (*p* = 0.064; Table 9 and Table S5). In the curative arm, the WO stage had significantly lower levels of this family than both TR and C stages of this arm (*p* ≤ 0.001 in each case; Table 9 and Table S5), and the TR stage of the curative arm was significantly different from the TR stage of the preventive arm (*p* = 0.008; Table 9 and Table S5), while not significantly different from the TR stage of the PPI control arm (*p* = 0.873; Table 9 and Table S5). This suggests that the decrease in *Streptococcaceae* was associated with probiotic treatment.

In the mucus of the distal colon, levels of *Streptococcaceae* decreased significantly in the stages that coincided with probiotic treatment, i.e., in the TR stage of the preventive arm compared with the C stage of the preventive arm (*p* = 0.013) and TR stage of the PPI control arm (*p* = 0.001), and in the WO stage of the curative arm compared with the C and TR stages of the curative arm (*p* = 0.001 in each case; Table 9 and Table S5) and the WO stages of the PPI control and preventive arms (*p* < 0.001 for each comparison; Table 9 and Table S5). This suggests that in this environment as well, *Streptococcaceae* levels decreased with probiotic treatment.

##### Changes in the Colonic Community: OTU-Level Shifts

The OTU closely related to *B. clausii* (OTU00031) was significantly enriched in the TR stage of the preventive arm (*p* = 0.001) and the WO stage of the curative arm (*p* = 0.002), compared with the C stage of the same arm, indicating a successful, albeit transient, engraftment of this species in the microbiota in all four colonic environments (Table 10 and Table S6).

In the lumen of the proximal colon, PPI treatment led to significant decreases in levels of the OTU related to *Bifidobacterium adolescentis* (OTU00017) in the TR stage of the PPI control arm compared with the C stage of the same arm (*p* = 0.038; Table 10 and Table S6). However, this decrease was also present in the preventive arm, indicating that probiotic treatment did not affect this OTU-level shift. Similarly, significant increases in the OTUs related to *E. faecalis*, *Anaerostipes hadrus* (significantly higher only in the preventive arm and numerically higher even though not statistically significant in the PPI control arm), and *Blautia obeum* were not affected by probiotic treatment (Table 10).

In the lumen of the distal colon, PPI treatment led to significant decreases in the levels of the OTU related to *Gemmiger formicilis* (OTU000120) in the TR stage of the PPI control arm compared with the C stage of the same arm (*p* = 0.017; Table 10 and Table S6). Probiotic treatment in the TR stage of the preventive arm maintained levels close to the C stage of this arm, while probiotic treatment in the curative arm significantly increased the levels of this species in the WO stage compared with both C and TR stages in this arm (*p* = 0.002 in each case; Table 10 and Table S6). Thus, probiotic treatment countered the PPI-induced reduction in the levels of this OTU.

Similar trends were observed in the lumen of the distal colon for the OTU related to *Akkermansia muciniphila* (OTU00036): the TR stage of the PPI control arm had significantly lower levels of this bacterium than the C stage (*p* = 0.03). In the preventive arm, even though numbers of this bacterium decreased in the TR stage, this was not statistically significant compared with the C stage (*p* = 0.062; Table 10 and Table S6). In the curative arm, probiotic treatment significantly increased levels in the WO stage compared to the TR stage of the same arm (*p* = 0.02; Table 10 and Table S6); however, these levels were comparable to the levels found in the WO stage of the PPI control arm (*p* = 0.172; Table 10 and Table S6). Thus, probiotic treatment maintained the levels of this OTU, which would have otherwise decreased due to PPI treatment.

There were significant increases in levels of the OTUs related to *A. hadrus* (OTU00018) in the TR stage of the PPI control and preventive arms compared with the C stage of the respective arms (*p* ≤ 0.001 in each case), and those of *B. obeum* (OTU00013) and *E. faecalis* (OTU00003) in the TR and WO stages of the PPI control, preventive, and curative arms compared with the C stages of the respective arms, indicating that probiotic treatment did not affect this OTU-level shift caused by PPI treatment (*p* < 0.05 in each case; Table 10 and Table S6).

In the mucus of the proximal colon, PPI treatment in the TR stage of the PPI control arm led to significant decreases (*p* < 0.001) compared to the C stage in the levels of the OTU related to *Prevotella denticola* (OTU00007), which was rescued by probiotic treatment in the preventive arm compared with the PPI control arm (*p* = 0.001; Table 10 and Table S6). Transient increases in the OTU related to *Streptococcus bovis* (OTU00001) in the TR stage of the PPI control arm were rescued by probiotic treatment, as seen by the lower levels of this OTU in the TR stage of the preventive arm and WO stage of the curative arm compared with the corresponding stages in the PPI control arm (Table 10). Increases in levels of the OTUs related to *A. hadrus* and *B. obeum* were further increased with probiotic treatment in the TR stage of the preventive arm compared with the C stage in the same arm and in the WO stage of the curative arms compared with the C and TR stages (*p* < 0.05 in each case; Table 10 and Table S6). Levels of *G. formicilis* which were significantly increased only in the WO stage of the PPI control arm saw further statistically significant increases in the preventive and curative arms compared to the C stages of these arms (*p* < 0.05 in each case; Table 10 and Table S6).

In the mucus of the distal colon, PPI treatment led to significant decreases in levels of OTUs related to *B. adolescentis* (OTU00017) (*p* = 0.001) and *Enterococcus faecium* (OTU00008) (*p* = 0.046) in the TR stage compared with the C stage of the PPI control arm (Table 10 and Table S6), which were not rescued by probiotic treatment in the preventive arm (Table 10). In the curative arm, levels of these OTUs were comparable between the WO and C stages. The significant increase in levels of *B. obeum* in the TR (*p* = 0.005) and WO (*p* = 0.002) stages of the PPI control arm compared with the C stage (Table 10 and Table S6) saw a further increase with probiotic treatment, with statistically increased levels in the TR stage of the preventive arm (*p* = 0.002) and WO stage of the curative arms (*p* = 0.031) compared with the C stages of the respective arms (Table 10 and Table S6). The significantly higher levels of the OTU related to *G. formicilis* in the WO stage compared to the C stage of the PPI control arm (*p* = 0.047) were also seen in the preventive (*p* = 0.001) and curative (*p* = 0.002) arms (Table 10 and Table S6).

#### 3.3.3. Overall Fermentative Activity

The pH of the SHIME system is tightly controlled by a pH controller to ensure that optimal conditions are maintained in the different reactors. However, when exposed to different treatments, the bacteria in the reactors may produce higher levels of acids, especially SCFA. As a result, the environment in the reactors becomes more acidic, requiring the addition of a base to maintain the respective optimal pH ranges. Even though not all acids produced by microbial fermentation led to a change in pH, measuring the acid–base consumption during the course of an investigation allows us to estimate the effect of the treatment on the overall microbial fermentative activity.

In the ileum, PPI treatment increased the acidity in all arms, possibly due to the additional administration of sugars and proteins (Table S7). In the proximal colon, there was a significant increase in acidity in the TR stage of the preventive arm compared with the TR stage of the PPI control arm (*p* < 0.001) and curative arm (*p* = 0.027). In the distal colon, the WO stage of the curative arm showed a significant increase in acidity when compared to the WO stage of the PPI control (*p* = 0.027). This indicates that in the colonic environments, probiotic treatment leads to an increase in acidity, likely due to increased production of acidic metabolites.

#### 3.3.4. Microbial Community Activity

**Figure 4:**
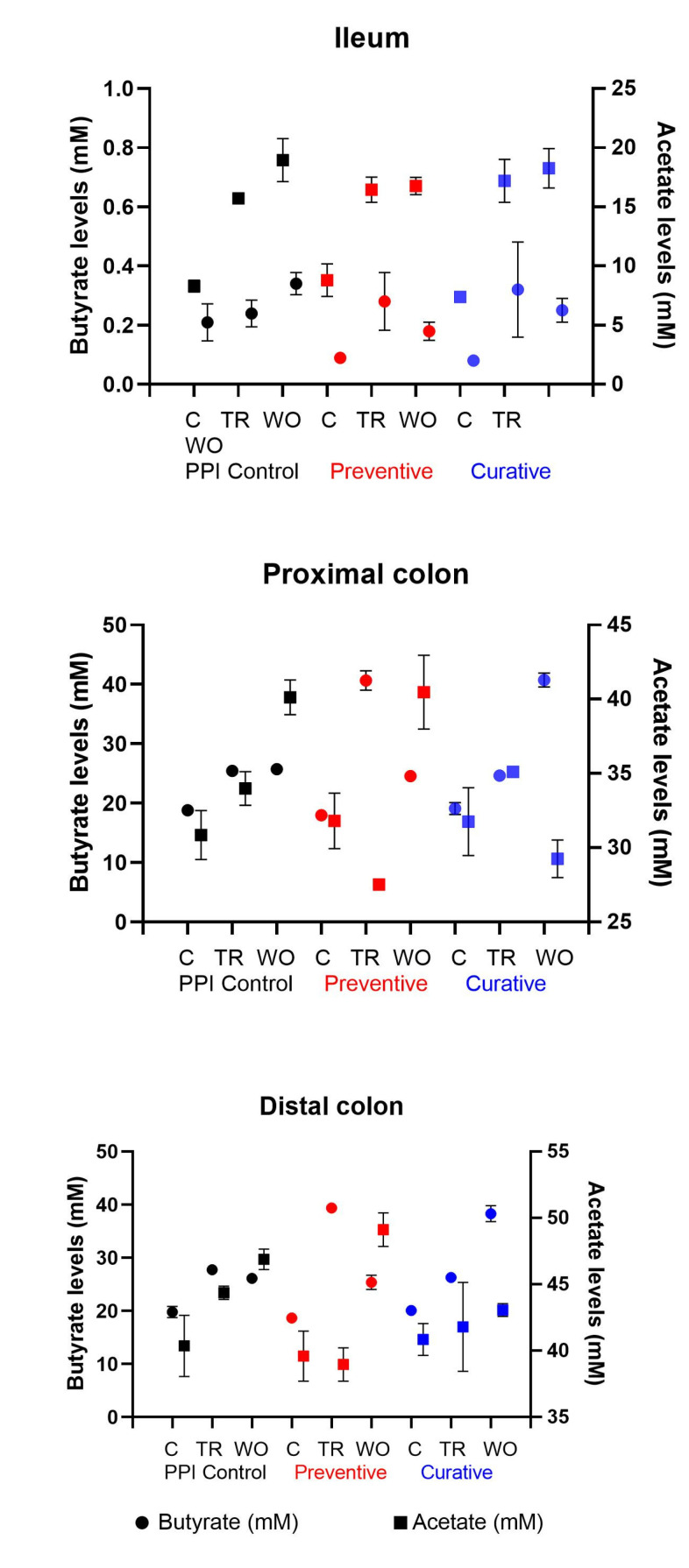
Butyrate production in the different arms of the study. Mean ± SD of six biological replicates for C (three replicates in week 1 and three replicates in week 2) and three biological replicates for TR and WO (three replicates in week 2) are shown.

We evaluated the levels of butyrate, acetate, propionate, and lactate as a measure of carbohydrate metabolism. In the ileum, butyrate levels were lower and had larger standard deviations than in the proximal and distal colon, possibly due to low numbers of butyrate producers and the low retention time in this reactor selecting against butyrate production (Figure 4).

PPI treatment led to significant increases in butyrate levels in the TR stage in all arms (*p* < 0.004 in each case, Table S8) with the exception of the PPI control in the ileum. In the proximal and distal colon, butyrate levels increased significantly in the TR stage of the preventive arm and the WO stage of the curative arm compared with the TR stage of the respective PPI control arms (*p* < 0.001 in each case, Table S8), indicating that butyrate levels increased further in response to probiotic treatment (Figure 4).

There was an inverse association between acetate levels and butyrate levels in the TR and WO stages of the preventive arm in the proximal and distal colon (Figure 4); this may indicate that acetate is converted to butyrate in these environments. This change was also transient, as seen in the WO stages of the preventive arm in the proximal and distal colon (Figure 4). A similar trend was seen in the WO stage of the curative arm (Figure 4).

Propionate, lactate, ammonium, and SCFA levels were evaluated in all arms; however, the increases in their levels were attributed to the additional sugars and proteins added to the SHIME medium rather than due to the treatments, due to their sustained increased levels in the TR and WO stages in all arms (Tables S9–S12). Thus, in this study, the main effect of the Enterogermina appears to be on acetate and butyrate levels.

## 4. Discussion

The healthy adult intestinal microbiota is dominated by the Bacteroidetes and Firmicutes phyla and is relatively stable with a core of ~40 species [28]. During acute perturbations, such as antibiotic treatment, the microbiota can be depleted to a community with low diversity [29]. However, after a period of recovery, microbial communities recover to their pre-treatment states, although this recovery may be incomplete [29]. However, chronic perturbations, such as those due to the long-term use of PPIs, may lead to more profound alterations in the gut microbiome. In a population-based study that compared the gut microbiota of long-term PPI users with those of non-users, there were significant differences in the relative abundances of certain bacterial species. Notably, the ratio of bacteria belonging to Firmicutes to those belonging to Bacteroides was altered in long-term PPI users, with specific increases in the abundance of Firmicutes species belonging to *Holdemania*, *Streptococcus,* and *Blautia* genera [30]. Our study also demonstrated an increase in Firmicutes levels with PPI treatment, with specific increases in *B. obeum* and *S. bovis* (Table 10). This indicates that the triple M-SHIME model can be used successfully to replicate some, if not all, effects of long-term PPI use in humans. In addition, our study has shown that some of these effects can be countered using probiotics—indicated by the reduction in *S. bovis* levels following probiotic treatment. Furthermore, because *S. bovis* is implicated in colorectal cancer [31,32], future research should investigate the promising role of *B*. *clausii* treatment in reducing the colon levels of this species.

Butyrate is an SCFA that is often associated with positive health outcomes. It is the primary energy source of intestinal epithelial cells, and it affects gut motility, regulates gut endocrine functions, promotes gut epithelial barrier functions by reducing epithelial permeability, and improves gut immune responses [33]. The increase in butyrate levels with probiotic treatment may indicate that the probiotic alters the gut microbiome environment in a way that potentially benefits the overall gastrointestinal health of the host.

Probiotic bacteria can modulate the fermentation processes in the gut directly through the production of metabolites or indirectly by generating metabolic end-products that can be used as cross-feeding substrates by other gut bacteria; this can lead to changes in the composition of the gut microbiota [34]. However, such processes are difficult to examine via in vivo models. Likewise, the results from clinical trials often do not examine nor determine the intermediate physiological processes that happen in order to achieve the outcomes they report. Animal models are also limited by their differences from humans in their normal colonic microbiota and the physiology of their gastrointestinal tract (e.g., differences in pH, retention time, bile salt levels, and temperature). On the other hand, in vitro models that accurately simulate the human gastrointestinal tract allow the sampling of each region without the need for invasive methods.

The SHIME model is an in vitro model that has been used for over 25 years and has been validated with in vivo parameters [35]. We have used the Triple-M-SHIME model, with a novel ileal compartment, to replicate the distinct microbial habitats that are present in the human gastrointestinal tract [28]. The Triple-M-SHIME can be used to localize the changes in microbial community composition and activity following controlled perturbations, such as PPI or probiotic treatment.

Though providing more insights into the mechanisms of *B. clausii*, this study still has some limitations. The tested probiotic formulation contains excipients, of which the combined amount of xylitol and microcrystalline cellulose exceeds 500 mg per 2 g sachet. Therefore, we cannot exclude the possibility that the excipients may have affected the results. The Triple-M-SHIME reactor with an added ileal compartment is a novel model of PPI dysbiosis, and further testing and validation will be needed to establish this as a robust representation of PPI dysbiosis in the real world. In addition, the donor whose faecal sample was used to inoculate the colonic reactors was not a long-term PPI user; therefore, changes arising from chronic PPI use may not have been accurately represented in this model. Since the donor was pre-selected to have higher levels of *Enterobacteriaceae*, the colonic community and changes observed may therefore not be generalizable to a broader population. Finally, an important limitation is that this was an exploratory study that aimed to have a global view of the changes arising from PPI use and probiotic treatment at different time points. There were no hypotheses concerning the direction or magnitude of changes to the composition or activity of the gut microbiota with PPI or probiotic treatment, and our study did not include an arm for pre-treatment with probiotic prior to PPI administration. Therefore, the conclusions arising from the statistical comparisons in this study need to be interpreted as preliminary findings that remain to be confirmed in future studies. We propose below some hypotheses based on our findings that may be tested in future research into the effects of PPI use on the gut microbiota and the effect of probiotics in this environment.

### Post Hoc Hypotheses Generated in this Study

**Figure 5:**
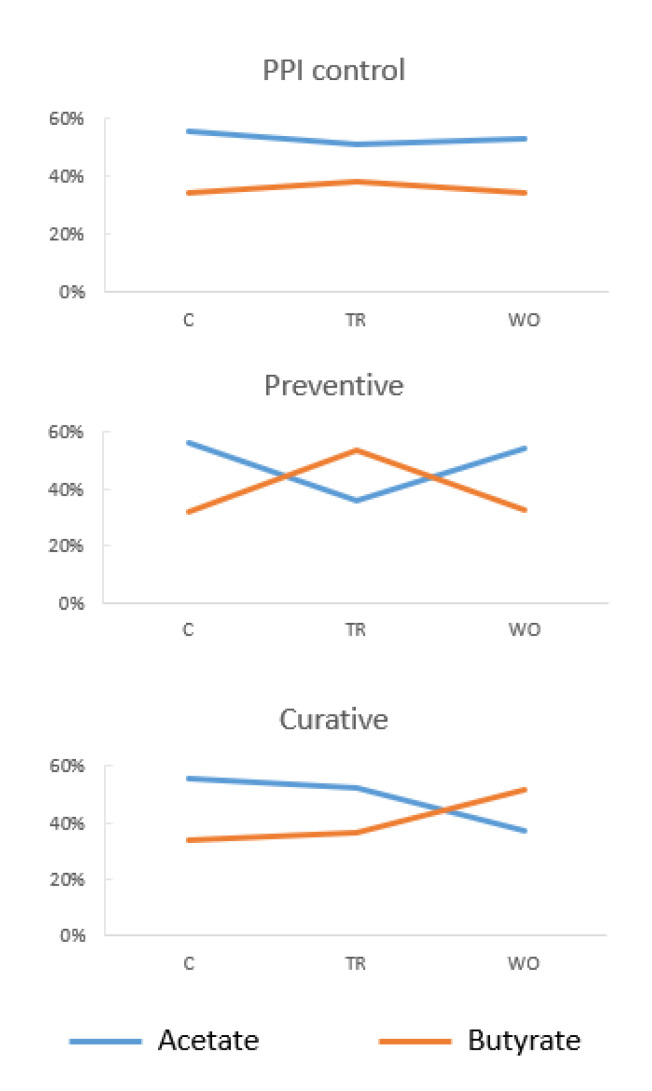
Proposed effect of Enterogermina on SCFA levels in the proximal colon: butyrate levels are inversely correlated with acetate levels in the presence of the probiotic. C, control stage; PPI, proton-pump inhibitor; TR, treatment stage; WO, washout stage.

This was an exploratory study with no prior hypotheses regarding the mechanism of action of *B. clausii* in tackling PPI-induced dysbiosis. Our analyses of the data allow us to generate post hoc hypotheses that can be tested in future appropriately powered studies, considering the effect sizes observed in this exploratory study. Below we list some of the potential avenues for future research based on the hypotheses generated in this study:

*B. clausii* treatment can mitigate PPI-induced dysbiosis by:

- Increasing the microbial diversity of colonic environments.
- Countering the PPI-induced decrease in the levels of *Coriobacteriaceae*, *Selenomonadaceae,* and *Akkermansiaceae* in the lumen of the distal colon, especially by maintaining levels of *G. formicilis* and *A. muciniphila*.
- Countering the PPI-induced increase in levels of *Streptococcaceae*, specifically *S. bovis*, in the mucus of the proximal colon.
- Maintaining levels of *P. denticola* in the mucus of the proximal colon.
- Increasing butyrate levels in the colon through aiding cross-feeding interactions that convert acetate into butyrate (Figure 5) and increasing levels of butyrate producers (such as *G. formicilis*).

## 5. Conclusions

Probiotic treatment with *B. clausii (O/C, N/R, SIN, T)*, either simultaneously with PPIs or immediately after use, has beneficial effects on the gut microbiota, as seen by the increase in microbial diversity and butyrate production following probiotic treatment. These conclusions are based on the use of the specific Triple-M-SHIME in vitro model of PPI dysbiosis used in this study, although the robustness of our findings need to be validated in future studies. To this end, we have proposed several hypotheses regarding the mechanism of action of *B. clausii* in countering PPI-induced dysbiosis based on our exploratory analysis of the changes to gut microbiota composition and function.

## Figures and Tables

**Table 1:** Primer sequences.

Primer	Sequence
*erm34* forward primer	AATTTTYACCGCCCCTCAAG
*erm34* reverse primer	AYTTTTGGAACATGCCGAAC
16S forward primer	CCTACGGGNGGCWGCAG
16S reverse primer	GACTACHVGGGTATCTAAKCC

**Table 2:** Average abundance of different OTUs (log cells/mL) in the lumen of the ileum in the different arms of the study. Means of three biological replicates are shown for each stage. The colour gradient correlates with the abundance, and is normalised for each OTU, i.e., each row. C, control; LOQ, limit of quantification; OTU, operational taxonomic unit; PPI, proton-pump inhibitor; TR, treatment; WO, washout.

		PPI Control			Preventive			Curative		
OTU	Closely-Related Species	C	TR	WO	C	TR	WO	C	TR	WO
OTU00031	*Bacillus clausii*	<LOQ	<LOQ	<LOQ	<LOQ	7.07	6.3	<LOQ	<LOQ	7.54
OTU00043	*Clostridium nexile*	6.62	6.95	7.03	6.69	6.88	7.26	6.64	6.78	7.41
OTU00003	*Enterococcus faecalis*	7.28	8.46	8.55	7.2	8.51	8.7	6.73	8.27	8.73
OTU00008	*Enterococcus faecium*	8.31	8.1	8.18	8.1	8.2	8.31	8.02	8.02	8.56
OTU00020	*Lactobacillus reuteri*	6.43	7.12	7.37	6.41	7.64	7.89	6.27	6.9	7.98
OTU00058	*Lactobacillus salivarius*	6.88	6.8	6.76	6.51	6.6	6.7	5.76	6.03	6.63
OTU00005	*Faecalibacterium prausnitzii*	<LOQ	<LOQ	<LOQ	<LOQ	<LOQ	<LOQ	<LOQ	<LOQ	<LOQ
OTU00001	*Streptococcus bovis*	9.29	9.17	8.9	9.23	9.1	8.89	9.15	9.3	8.68
OTU00064	*Streptococcus intermedius*	7.38	6.05	6.43	6.02	5.69	6.23	5.64	5.98	5.6
OTU00002	*Veillonella parvula*	8.1	8.22	8.52	8.21	8.5	8.64	8.14	8.5	8.79
OTU00182	*Veillonella dispar*	<LOQ	<LOQ	<LOQ	<LOQ	5.41	<LOQ	<LOQ	5.47	<LOQ
		Lower levels of the OTU			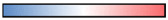			Higher levels of the OTU		

**Table 3:** Average abundance of different OTUs (relative abundance in %) in the mucus of the ileum in the different arms of the study. Means of three biological replicates are shown for each stage. The colour gradient correlates with the abundance and is normalised for each OTU, i.e., within each row. C, control; OTU, operational taxonomic unit; PPI, proton-pump inhibitor; TR, treatment; WO, washout.

		PPI Control			Preventive			Curative		
OTU	Closely-Related Species	C	TR	WO	C	TR	WO	C	TR	WO
OTU00031	*Bacillus clausii*	0	0.08	0	0	0.59	0.19	0	0	5.15
OTU00043	*Clostridium nexile*	0.17	0.73	0.29	0.09	0.18	0.18	0.12	0.48	1.05
OTU00003	*Enterococcus faecalis*	0.45	6.38	18.97	0.34	7.65	29.18	0.16	11.34	20.84
OTU00008	*Enterococcus faecium*	3.63	9.69	10.87	3.52	10.18	7.95	2.59	7.27	18.05
OTU00020	*Lactobacillus reuteri*	0.13	1.04	2.33	0.18	1.25	3.09	0.14	0.86	2.11
OTU00058	*Lactobacillus salivarius*	0.17	0.35	0.25	0.03	0.06	0.07	0.01	0.07	0.09
OTU00005	*Faecalibacterium prausnitzii*	0.01	0	0	0	0	0	0	0.01	0
OTU00001	*Streptococcus bovis*	91.65	60.63	34.22	93.93	46.67	31.74	92.92	65.79	10.52
OTU00064	*Streptococcus intermedius*	0.71	0.24	0.24	0.02	0.05	0.07	0.01	0.05	0.02
OTU00002	*Veillonella parvula*	3.08	20.82	27.05	1.82	33.3	24.17	4.04	14.1	28.65
OTU00183	*Veillonella dispar*	0	0.01	0.01	0	0	0	0.01	0	0
		Lower levels of the OTU			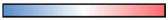			Higher levels of the OTU		

**Table 4:** Reciprocal Simpson diversity index in the lumen and mucus of the proximal and distal colon. Means of three biological replicates are shown. The colour gradient represents the gradient in the absolute diversity index and is normalised for each row. C, control; DC, distal colon; OTU, operational taxonomic unit; PC, proximal colon; PPI, proton-pump inhibitor; TR, treatment; WO, washout.

		PPI Control			Preventive			Curative		
		C	TR	WO	C	TR	WO	C	TR	WO
Lumen	PC	5.3	4.1	12.6	4.7	7.1	12.7	5.6	5.4	8.4
	DC	4.7	3.1	15.4	3.6	7	14.2	3.6	4.2	13.3
Mucus	PC	9.7	7.3	7.3	9.1	11.2	10.6	7.9	7.9	9.1
	DC	7.8	8.6	18.3	10.1	19.3	22.6	7.3	9.1	26.5
		Lower diversity			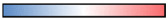			Higher diversity		

**Table 5:** Average levels (log cells/mL) of different phyla in the lumen of the proximal colon. Means of three biological replicates are shown. The colour gradient correlates with the absolute levels of each phylum and is normalised within each row. C, control; DC, distal colon; LOQ, limit of quantification; OTU, operational taxonomic unit; PC, proximal colon; PPI, proton-pump inhibitor; TR, treatment; WO, washout.

	PPI Control			Preventive			Curative		
	C	TR	WO	C	TR	WO	C	TR	WO
Actinobacteria	8.91	8.84	9.05	8.81	8.72	8.97	8.96	8.89	8.78
Bacteroidetes	8.87	8.52	8.83	8.91	9.13	9	8.65	8.65	9.36
Desulfobacteria	<LOQ	<LOQ	6.05	<LOQ	<LOQ	<LOQ	<LOQ	<LOQ	<LOQ
Firmicutes	9.48	9.53	9.61	9.58	9.6	9.62	9.49	9.62	9.73
Proteobacteria	<LOQ	<LOQ	<LOQ	<LOQ	<LOQ	5.89	<LOQ	<LOQ	6.21
Synergistetes	<LOQ	<LOQ	6.54	<LOQ	<LOQ	6.91	<LOQ	6.38	6.33
	Lower levels of the phylum			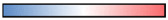			Higher levels of the phylum		

**Table 6:** Average levels (log cells/mL) of different phyla in the lumen of the distal colon. Means of three biological replicates are shown. The colour gradient correlates with the absolute levels of each phylum and is normalised within each row. C, control; PPI, proton-pump inhibitor; TR, treatment; WO, washout.

	PPI Control			Preventive			Curative		
	C	TR	WO	C	TR	WO	C	TR	WO
Actinobacteria	9.09	8.83	9.19	8.84	8.82	9.01	9.33	8.95	8.8
Bacteroidetes	8.79	8.59	9.15	8.94	8.93	9.16	9.11	8.69	9.36
Desulfobacteria	7.17	7.22	7.44	7	7.4	7.36	7.21	7.15	7.85
Firmicutes	9.46	9.58	9.58	9.52	9.51	9.49	9.7	9.63	9.65
Proteobacteria	7.14	6.88	7.29	6.88	7.16	7.18	6.96	7.09	7.5
Synergistetes	5.92	<LOQ	7.12	5.94	6.53	6.82	<LOQ	6.62	6.85
Verrucomicrobia	7.71	7.09	7.97	7.85	7.33	7.6	7.78	7.08	8.19
	Lower levels of the phylum			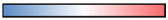			Higher levels of the phylum		

**Table 7:** Average abundance (%) of different phyla in the mucus of the proximal colon. Means of three biological replicates are shown. The colour gradient correlates with the relative levels of each phylum and is normalised within each row. C, control; PPI, proton-pump inhibitor; TR, treatment; WO, washout.

	PPI Control			Preventive			Curative		
	C	TR	WO	C	TR	WO	C	TR	WO
Actinobacteria	22.7%	27.5%	24.9%	17.8%	20.8%	20.1%	19.0%	21.1%	16.8%
Bacteroidetes	10.0%	5.3%	8.6%	9.3%	10.6%	10.3%	9.1%	7.3%	11.2%
Desulfobacteria	0.0%	0.1%	0.0%	0.0%	0.0%	0.0%	0.0%	0.0%	0.0%
Firmicutes	67.3%	67.1%	66.2%	72.9%	68.6%	69.1%	72.0%	71.4%	71.8%
Synergistetes	0.0%	0.0%	0.3%	0.0%	0.0%	0.4%	0.0%	0.3%	0.2%
	Lower levels of the phylum			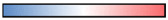			Higher levels of the phylum		

**Table 8:** Average abundance (%) of different phyla in the mucus of the distal colon. Means of three biological replicates are shown. The colour gradient correlates with the relative levels of each phylum and is normalised within each row. C, control; PPI, proton-pump inhibitor; TR, treatment; WO, washout.

	PPI Control			Preventive			Curative		
	C	TR	WO	C	TR	WO	C	TR	WO
Actinobacteria	16.4%	19.0%	25.5%	12.4%	15.9%	18.0%	21.2%	18.8%	18.6%
Bacteroidetes	9.5%	11.5%	15.0%	13.8%	14.1%	18.6%	10.2%	11.6%	19.8%
Desulfobacteria	1.3%	1.2%	1.0%	1.0%	1.3%	1.2%	0.7%	0.9%	1.8%
Firmicutes	71.3%	66.7%	56.3%	71.5%	66.6%	60.1%	66.9%	67.3%	56.2%
Proteobacteria	0.4%	0.8%	0.9%	0.2%	0.9%	0.8%	0.4%	0.5%	1.8%
Synergistetes	0.1%	0.2%	0.5%	0.1%	0.5%	1.0%	0.1%	0.5%	0.6%
Verrucomicrobia	1.0%	0.5%	0.7%	1.0%	0.6%	0.5%	0.5%	0.3%	1.1%
	Lower levels of the phylum			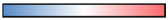			Higher levels of the phylum		

**Table 9:** Family-level shifts in the colonic communities in the different arms of the study. For the luminal environments, average levels (log cells/mL) of three biological replicates are shown. For the mucosal environments, average abundance (%) of three biological replicates are shown. The colour gradient correlates with the levels of each family and is normalised within each row. C, control; PPI, proton-pump inhibitor; TR, treatment; WO, washout.

			PPI Control			Preventive			Curative			
	Phylum	Family	C	TR	WO	C	TR	WO	C	TR	WO	
Proximal colon lumen	Bacteroidetes	*Bacteroidaceae*	8.1	7.42	8.35	8.15	7.25	8.47	8.2	8.09	7.57	
	Firmicutes	*Bacillaceae*	<LOQ	<LOQ	6.27	<LOQ	7.01	6.52	<LOQ	<LOQ	7.08	
		*Ruminococcaceae*	8.64	8.84	9.21	8.75	9	9.25	8.7	8.91	9.33	
Distal colon lumen	Actinobacteria	*Coriobacteriaceae*	6.6	6.28	6.8	6.65	6.49	6.59	6.46	6.6	6.44	
	Firmicutes	*Christensenellaceae*	6.43	6.37	7.06	6.27	7.04	6.84	6.03	6.75	7.4	
		*Enterococcaceae*	7.68	7.82	8.43	7.58	7.98	8.65	7.52	7.94	8.61	
		*Selenomonadaceae*	7.3	6.89	7.42	7.5	7.15	7.3	7.46	7.14	7.11	
	Verrucomicrobia	*Akkermansiaceae*	7.71	7.09	7.97	7.85	7.33	7.6	7.78	7.08	8.19	
Proximal colon mucus	Bacteroidetes	*Prevotellaceae*	8.60%	3.60%	6.00%	7.90%	9.70%	6.30%	8.10%	5.10%	9.00%	
		*Streptococcaceae*	17.60%	28.70%	6.30%	21.10%	14.50%	3.30%	21.40%	25.80%	3.10%	
Distal colon mucus	Bacteroidetes	*Streptococcaceae*	33.10%	30.80%	13.20%	28.50%	16.30%	11.40%	34.00%	29.50%	3.50%	
			Lower levels of the family			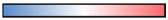			Higher levels of the family		

**Table 10:** OTU-level shifts in the colonic communities in the different arms of the study. For the luminal environments, average levels (log cells/mL) of three biological replicates are shown. For the mucosal environments, average abundance (%) of three biological replicates are shown. The colour gradient correlates with the absolute levels of each OTU and is normalised within each row. C, control; PPI, proton-pump inhibitor; TR, treatment; WO, washout.

			PPI Control			Preventive			Curative			
Stage	OTU	Closely-Related Species	C	TR	WO	C	TR	WO	C	TR	WO	
Proximal colon lumen	OTU00017	*Bifidobacterium adolescentis*	8.12	7.72	8.19	8.24	7.41	7.73	7.67	7.64	7.83
	OTU00003	*Enterococcus faecalis*	6.99	7.98	8.4	7.04	8.24	8.65	6.46	7.95	8.53
	OTU00018	*Anaerostipes hadrus*	<LOQ	6.44	6.38	<LOQ	8.38	6.52	6.16	<LOQ	8.83
	OTU00013	*Blautia obeum*	6.67	7.5	7.83	6.94	8.31	7.66	6.9	7.78	8.69
Distal colon lumen	OTU00003	*Enterococcus faecalis*	6.6	7.56	8.35	6.64	7.75	8.61	6.16	7.66	8.49
	OTU00018	*Anaerostipes hadrus*	<LOQ	6.6	6.09	<LOQ	8.45	6.15	<LOQ	<LOQ	8.77
	OTU00013	*Blautia obeum*	6.29	7.35	7.85	6.66	7.88	7.57	6.57	7.66	8.74
	OTU00012	*Gemmiger formicilis*	7.84	7.47	8.44	7.72	8.01	8.54	7.7	7.5	8.71
	OTU00036	*Akkermansia muciniphila*	7.71	7.09	7.97	7.85	7.33	7.6	7.78	7.08	8.19
Proximal colon mucus	OTU00004	*Bifidobacterium dentium*	10.10%	14.70%	13.40%	2.70%	11.80%	10.30%	10.20%	14%	5.20%
	OTU00007	*Prevotella denticola*	8.30%	3.10%	5.30%	7.70%	7.60%	5.70%	8%	4.50%	7.70%
	OTU00018	*Anaerostipes hadrus*	0	0.10%	0	0	2.90%	0.10%	0	0	5.40%
	OTU00013	*Blautia obeum*	0.20%	1.60%	1.20%	0.50%	9.90%	2.20%	0.30%	1.50%	7.70%
	OTU00012	*Gemmiger formicilis*	0.60%	0.60%	2.20%	0.50%	1.60%	5.30%	0.30%	0.70%	3.70%
	OTU00001	*Streptococcus bovis*	17.40%	28.60%	6.30%	21%	14.40%	3.30%	21.30%	25.80%	3.10%
Distal colon mucus	OTU00017	*Bifidobacterium adolescentis*	1.20%	0.20%	0.60%	0.70%	0.10%	0.20%	0.50%	0.20%	0.40%
	OTU00008	*Enterococcus faecium*	1.40%	0.70%	0.40%	1.40%	0.80%	0.20%	0.80%	0.80%	0.60%
	OTU00013	*Blautia obeum*	0.10%	1.80%	2.10%	0.40%	4.30%	1.90%	0.10%	1.70%	9.30%
	OTU00012	*Gemmiger formicilis*	0.40%	0.50%	0.90%	0.30%	1.10%	2.20%	0.20%	0.60%	1.60%
			Lower levels of the OTU			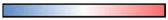			Higher levels of the OTU		

## Data Availability

Qualified researchers may request access to data and related study documents including the study report, study protocol with any amendments, statistical analysis plan, and dataset specifications. Further details on Sanofi’s data sharing criteria, eligible studies, and process for requesting access can be found at: https://www.vivli.org/ (accessed on 24 January 2023).

## Supplementary Materials

The following supporting information can be downloaded at: https://www.mdpi.com/article/10.3390/nu15030653/s1, Table S1: Comparisons between and within arms for species-specific qPCR to evaluate *B. clausii* levels; Table S2: Statistical comparisons of OTUs in ileum; Table S3: *p*-values of statistical comparisons for alpha diversity in treatment arms in the colon; Table S4: Phylum-level comparisons in the colon; Table S5: Family-level comparisons in the colon; Table S6: OTU-level comparisons in the colon; Table S7: Base-acid consumption; Table S8: *p*-values for statistical comparisons of butyrate levels and *p*-values for statistical comparisons of acetate levels; Table S9: Propionate levels: Table S10: *p*-values for statistical comparisons of ammonium levels; Table S11: Branched chain fatty acids (mM) and *p*-values for statistical comparisons of BCFA levels; Table S12: Lactate concentrations and *p*-values for comparison of lactate levels.
